# Interpretability-based machine learning for predicting the risk of death from pulmonary inflammation in Chinese intensive care unit patients

**DOI:** 10.3389/fmed.2024.1399527

**Published:** 2024-06-12

**Authors:** Yihai Zhai, Danxiu Lan, Siying Lv, Liqin Mo

**Affiliations:** Cardiothoracic Surgery Intensive Care Unit, The First Affiliated Hospital of Guangxi Medical University, Nanning, Guangxi, China

**Keywords:** intensive care unit, infection, mortality, machine learning, precision therapy

## Abstract

**Objective:**

The objective of this research was to create a machine learning predictive model that could be easily interpreted in order to precisely determine the risk of premature death in patients receiving intensive care after pulmonary inflammation.

**Methods:**

In this study, information from the China intensive care units (ICU) Open Source database was used to examine data from 2790 patients who had infections between January 2019 and December 2020. A 7:3 ratio was used to randomly assign the whole patient population to training and validation groups. This study used six machine learning techniques: logistic regression, random forest, gradient boosting tree, extreme gradient boosting tree (XGBoost), multilayer perceptron, and K-nearest neighbor. A cross-validation grid search method was used to search the parameters in each model. Eight metrics were used to assess the models’ performance: accuracy, precision, recall, F1 score, area under the curve (AUC) value, Brier score, Jordon’s index, and calibration slope. The machine methods were ranked based on how well they performed in each of these metrics. The best-performing models were selected for interpretation using both the Shapley Additive exPlanations (SHAP) and Local interpretable model-agnostic explanations (LIME) interpretable techniques.

**Results:**

A subset of the study cohort’s patients (120/1668, or 7.19%) died in the hospital following screening for inclusion and exclusion criteria. Using a cross-validated grid search to evaluate the six machine learning techniques, XGBoost showed good discriminative ability, achieving an accuracy score of 0.889 (0.874–0.904), precision score of 0.871 (0.849–0.893), recall score of 0.913 (0.890–0.936), F1 score of 0.891 (0.876–0.906), and AUC of 0.956 (0.939–0.973). Additionally, XGBoost exhibited excellent performance with a Brier score of 0.050, Jordon index of 0.947, and calibration slope of 1.074. It was also possible to create an interactive internet page using the XGBoost model.

**Conclusion:**

By identifying patients at higher risk of early mortality, machine learning-based mortality risk prediction models have the potential to significantly improve patient care by directing clinical decision making and enabling early detection of survival and mortality issues in patients with pulmonary inflammation disease.

## 1 Introduction

Worldwide, the incidence of infections in intensive care units (ICUs) surpasses that in general wards by approximately 5 to 10 times (1). Particularly prevalent among ICU patients are lower respiratory tract infections, which can constitute 40 to 50% of all infections (2, 3). Among these, lung inflammation is the most common respiratory disease ailment in the lower respiratory tract, contributing significantly to global mortality rates (4).

As the core organ of the respiratory system, impaired lung function can disrupt the balance of oxygen and carbon dioxide in the blood and cause a buildup of metabolic products. This can worsen the body’s physiological stress response and lead to serious complications such as acute respiratory failure and sepsis, significantly increasing the risk of death (5, 6). Notably, approximately 20 to 30% of patients with pneumonia admitted to the ICU die within 1 week (7). Thus, early detection of patients with inflammatory lung disease who are at high risk of death is crucial.

Current studies aiming to predict the probability of death in ICU patients encompass various factors, including cerebral infarction (8), acute heart failure (9), sepsis (10), healthcare-associated infections (HAIs) (11), and other domains. However, there have been few investigations on the risk of death from lung inflammation. Sepsis emerges as the most extensively studied area in ICU mortality risk research. Typically triggered by an underlying condition such as a lung infection, its presence indicates that the disease has progressed to a severe level. As a result, early detection of the onset and progression of pulmonary inflammation has major implications for optimizing therapy and improving patient outcomes.

Existing mortality risk models primarily use demographic data from patients outside of China, and Chinese patients are not adequately represented. This limits the ability of existing models to accurately forecast the probability of death in Chinese ICU patients. Hence, patients in China may differ significantly from those in other countries in terms of demographics, disease spectrum, medical procedures, and lifestyle.

Today, determining a patient’s risk of death is a challenging clinical task. Machine learning emerges as a potential approach for identifying this risk (12), capable of capturing complex non-linear relationships to accurately identify patterns and features associated with the risk of death by learning from a large amount of clinical data and biochemical indicator data, allowing physicians to make more accurate diagnostic and therapeutic decisions (13).

The goal of this study was to create and verify an interpretable machine learning-based mortality risk prediction model for Chinese ICU patients with pulmonary inflammatory illness. It provides guidance to healthcare practitioners by exploring in-depth the risk factors associated with death. By identifying unfavorable patient outcomes in the early stages of the disease, timely intervention can be implemented, leading to improved patient survival and ultimately enhancing clinical decision making and patient outcomes.

## 2 Materials and methods

### 2.1 Study population and outcome

The data used in this study to estimate the probability of death in patients with pulmonary inflammation were obtained from the Critical Care Database version 1.1. This database is an open-source database for intensive care units in Zigong City, Sichuan Province, China, and specifically contains patients with infection (14). The Ethics Committee of the Fourth People’s Hospital in Zigong approved the use of this data (Ethics Approval No. 2020-065). The database includes information from 2790 infected individuals (excluding those with COVID-19 pneumonia), such as laboratory test results, baseline characteristics, medication use records, International Classification of Diseases (ICD) codes, nursing records, and follow-up information.

**FIGURE 1:**
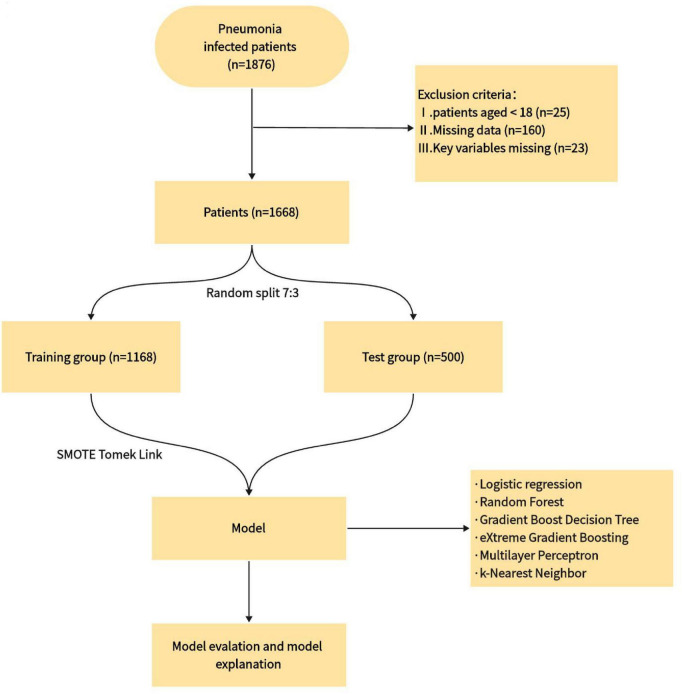
Patient selection flowchart and study design routes.

The inclusion criteria for this study were as follows: (1) age ≥18 years old and (2) infection site identified as “lung” according to ICD codes. The exclusion criteria were: (1) missing data values >25% and (2) missing key variables. A total of 1668 cases were included in the analysis. The patients were divided into two groups: Survivors and Non-survivors, based on their deceased or alive status. The study’s results were reported following the criteria for developing and publishing machine learning predictive models in biomedical research (15). Figure 1 illustrates the flowchart for the patients included in this study and the study design.

### 2.2 Variable selection and pre-processing

This study selected variables that reflect the disease and treatment effects based on clinical experience and database characteristics, including:

(1) General information: gender, age, history of chronic pulmonary disease, and history of diabetes mellitus;
(2) vital signs: diastolic blood pressure, systolic blood pressure, body temperature, respiration, heart rate, and type of respiratory support;
(3) laboratory tests: oxygen saturation in arterial blood (SaO2), white blood cell, albumin, blood creatinine, sodium ions, calcium ions, potassium ions, platelets, Alanine amioTransferase (ALT), Aspartate Aminotransferase (AST), hemoglobin (Hg), activated partial thromboplastin time (APTT), serum total bilirubin, high-sensitivity troponin-i (Tn-i), and international normalized ratio (INR). In total, 25 variables were included.

All variables were checked for outliers and missing values. Missing values greater than 25% were removed, while those less than 25% were addressed using multiple interpolations with the “mice” package in R. Additionally, all variables were mean standardized. Gender, history of chronic obstructive pulmonary disease, history of diabetes, and type of respiratory support were considered discrete variables, while the rest were considered continuous variables. Positive events are represented by a variable value of 1, while negative events are denoted by 0. Vital signs were also selected as the first recorded data upon ICU admission. Supplementary File 1 provides further details.

### 2.3 Sample equalization processing

The overall mortality rate at discharge in this trial was 7.19%, with a positive-to-negative ratio of approximately 1 to 13. In supervised learning, classification algorithms whose learning goal is overall classification accuracy tend to focus too much on the majority class and fail to learn characteristics from the minority class. To ensure the efficiency of machine learning, this study utilized the SMOTE Tomek Link algorithm, which combines oversampling and undersampling (16). This approach removes noise from samples and balances the sample size.

### 2.4 Model construction

#### 2.4.1 Machine learning model

In this study, Python software (version 3.10) was used to process the data. Logistic regression (LR), random forest (RF), gradient boost decision tree (GBDT), extreme gradient boosting tree (XGBoost), multilayer perceptron (MLP), and k-nearest neighbor (KNN) algorithms were used to predict the risk of death in patients with pneumonia.

#### 2.4.2 Model training

The dataset was divided into training and test sets in a 7:3 ratio. To improve the model’s generalization ability, 10-fold cross-validation was applied to the test set, and the model’s hyperparameters were adjusted using the GridSearchCV method. The model’s accuracy was estimated by averaging the data in the test set along with its 95% confidence interval. Eight metrics were used to evaluate the model outcomes: accuracy, precision, sensitivity, F1 score, area under the curve, Brier score, Jordan’s index, and calibration slope. Due to minimal variations in the performance metrics among most of the machine learning models, selecting the final model posed a challenge (17). In this study, each measure (such as accuracy, precision) was evaluated from highest to lowest and given a score ranging from 6 to 1, all the points are added together to make the total score. Therefore, The model with the highest score was chosen for further model interpretation.

#### 2.4.3 Model interpretability and variable importance

Variable importance was assessed using the Shapley Additive exPlanations (SHAP) method. For each predicted sample, the model generates a predicted value, and the SHAP value is the value assigned to each feature in that sample (18). SHAP allows for a global evaluation of the model by determining the marginal contribution of features to the model output. Complementing the SHAP method, the Local Interpretable Model-Agnostic Explanations (LIME) method improves the interpretability of the best model and its transparency in clinical practice (19). LIME calculates the risk of premature death and assigns individual weights to each variable, helping to understand changes in estimated probabilities under different observation settings and making the model more distinct.

### 2.5 Dataset description

The count data in the baseline data are expressed as frequencies and percentages, while the measurement data are expressed as mean ± standard deviation or median (interquartile range), depending on the numerical distribution. The appropriate statistical tests (*t*-test/Chi-square test/non-parametric tests) were used according to the data distribution shape, with α = 0.05.

## 3 Results

The average age of the patients was 67.55 ± 16.37 years, 17.03% had diabetes, and 14.09% had chronic lung disease. There were statistical differences in gender, temperature, systolic blood pressure, diastolic blood pressure, SaO_2_, type of respiratory support, APTT, albumin, AST, calcium ions, Tn-i, INR, and white blood cells between surviving and deceased patients. The other characteristics did not show statistical significance. The baseline characteristics of the dataset are summarized in Table 1. The “Total” category represents the information of the entire study population, including survivors and non-survivors groups.

**TABLE 1:** Comparison of various characteristics in the two groups of patients (*n* = 1668).

Characteristics	Total (*n* = 1668)	Survivors (*n* = 1548)	Non-survivors (*n* = 120)	*P*-value
Age (year)	67.55 ± 16.37	67.63 ± 16.20	66.45 ± 18.53	0.445
Gender				0.028
Male	1025 (61.45%)	940 (56.35%)	85 (5.10%)	
Female	643 (38.55%)	608 (36.45%)	35 (2.10%)	
Temperature (^°^F)	97.70 (97.16, 97.88)	97.70 (97.16, 98.06)	97.16 (96.80, 97.70)	0.001
Heart rate	98.00 (80.00, 118.00)	98.00 (80.00, 118.00)	98.50 (76.00, 121.00)	0.586
Systolic blood pressure (mmHg)	135.00 (110.00, 162.00)	136.00 (112.00, 163.00)	113.00 (150.75, 89.25)	<0.001
Diastolic blood pressure (mmHg)	80.00 (65.00, 95.00)	80.00 (66.00, 95.00)	72.00 (51.50, 91.50)	<0.001
SaO_2_ (%)	98.10 (96.80, 99.00)	98.20 (97.00, 99.00)	98.00 (95.10, 99.18)	<0.001
Respiratory rate	20.00 (16.00, 26.00)	20.00 (16.00, 26.00)	18.00 (15.00, 25.00)	0.195
Diabetes				0.913
Yes	284 (17.03%)	264 (15.83%)	20 (1.20%)	
No	1384 (82.97%)	1284 (76.98%)	100 (6.00%)	
Chronic pulmonary disease				0.766
Yes	235 (14.09%)	217 (13.01%)	18 (1.08%)	
No	1433 (85.91%)	1331 (79.80%)	102 (6.12%)	
Type of respiratory support				0.002
Invasive	499 (29.90%)	448 (26.85%)	51 (3.05%)	
Non-invasive	1169 (70.10%)	1100 (65.96%)	69 (4.14%)	
Activated partial thromboplastin time	29.00 (25.50, 33.10)	28.80 (25.40, 32.70)	31.80 (26.43, 44.80)	<0.001
Alanine aminotransferase	24.30 (15.30, 43.55)	24.00 (15.00, 42.00)	35.30 (19.78, 86.95)	0.053
Albumin	35.40 (30.13, 39.88)	35.50 (30.40, 39.98)	32.20 (27.15, 38.75)	0.003
Aspartate aminotransferase	32.55 (22.40, 61.08)	32.00 (21.93, 58.18)	51.80 (29.03, 96.15)	0.048
Calcium	2.19 (2.07, 2.31)	2.19 (2.08,2.31)	2.16 (2.01, 2.30)	0.046
Creatinine	71.55 (53.03, 104.53)	70.75 (52.73, 103.13)	78.75 (56.98, 126.65)	0.582
Hemoglobin	118.00 (99.00, 137.00)	118.00 (98.25, 137.00)	120.00 (100.50, 139.75)	0.535
High sensitivity troponin I	0.04 (0.01, 0.17)	0.03 (0.01, 0.16)	0.08 (0.02, 0.57)	<0.001
International normalized ratio	1.30 ± 0.53	1.27 ± 0.43	1.65 ± 1.23	<0.001
Platelet	147.00 (106.00, 204.00)	146.00 (106.00, 202.75)	153.50 (111.25, 121.75)	0.344
Potassium	3.58 (3.21, 4.08)	3.58 (3.21, 4.07)	3.54 (3.20, 4.20)	0.227
Sodium	138.65 (135.40,141.20)	138.70 (135.50,141.20)	138.05 (134.70,141.18)	0.740
Total bilirubin	13.70 (9.30, 20.30)	13.70 (9.30, 13.70)	13.90 (8.93, 21.25)	0.448
White blood cell	11.76 (7.99, 16.18)	11.60 (7.98, 16.05)	12.82 (8.30, 18.27)	0.014

The study adjusted some of the hyperparameters of the models using the GridSearchCV method, and the adjustment space and determined values of the hyperparameters can be found in Supplementary File 2. Table 2 displays the final 10-fold cross-validated model efficacy along with its 95% confidence intervals. In terms of individual model performance, the GBDT model has the highest accuracy, precision, F1-score, AUC value, Brier score, and Youden index, the KNN model had the highest recall, and the MLP model had the highest calibration slope. By calculating the distribution F1 value and AUC value (score = 0.6 F1 + 0.4 AUC), the optimal cutoff value for XGBoost was determined to be 0.510, achieving the highest score of 0.957.

**TABLE 2:** Predictive performance of six machine learning models.

Measure	LR	RF	GBDT	XGBoost	MLP	KNN
Accuracy	0.752 (0.727–0.777)	0.883 (0.852–0.914)	0.919 (0.901–0.937)	0.889 (0.874–0.904)	0.853 (0.821–0.886)	0.860 (0.836–0.883)
Precision	0.751 (0.723–0.780)	0.882 (0.848–0.917)	0.917 (0.895–0.939)	0.871 (0.849–0.893)	0.839 (0.806–0.871)	0.816 (0.793–0.840)
Recall	0.755 (0.712–0.799)	0.885 (0.856–0.915)	0.922 (0.894–0.951)	0.913 (0.890–0.936)	0.875 (0.831–0.918)	0.929 (0.901–0.957)
F1-score	0.752 (0.726–0.777)	0.883 (0.853–0.914)	0.919 (0.901–0.937)	0.891 (0.876–0.906)	0.855 (0.822–0.889)	0.869 (0.847–0.890)
AUC	0.822 (0.789–0.856)	0.955 (0.936–0.974)	0.971 (0.957–0.986)	0.956 (0.939–0.973)	0.920 (0.895–0.944)	0.873 (0.854–0.892)
Brier Score	0.170	0.064	0.032	0.050	0.061	0.112
Youden index	0.763	0.940	0.967	0.947	0.922	0.915
Calibration slope	0.961	1.302	0.957	1.074	0.985	0.886
Total score	13	28	45	36	25	22

**FIGURE 2:**
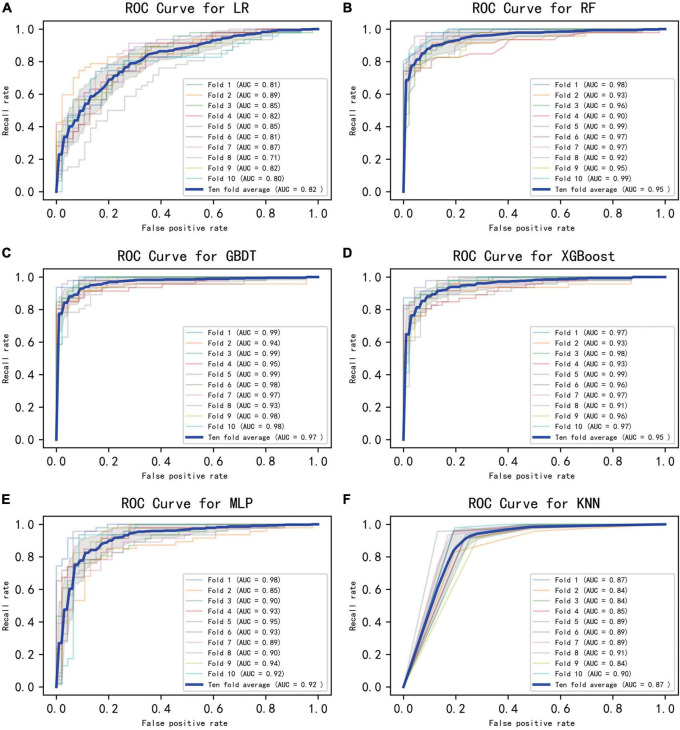
Ten-fold cross-validated ROC graphs for six learning models. **(A)** Logistic regression, **(B)** random forest, **(C)** gradient boosting decision tree, **(D)** XGBoost, **(E)** multilayer perceptron, **(F)** K-nearest neighbor. The shaded area in the figure represents the 95% confidence interval.

**FIGURE 3:**
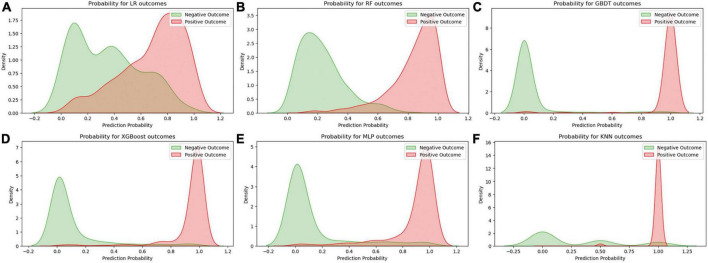
Predicted probability curves for the six learning models. **(A)** Logistic regression, **(B)** random forest, **(C)** gradient boosting decision tree, **(D)** XGBoost, **(E)** multilayer perceptron, **(F)** K-nearest neighbor. The green curve indicates patient survival, and the red curve indicates patient death.

**FIGURE 4:**
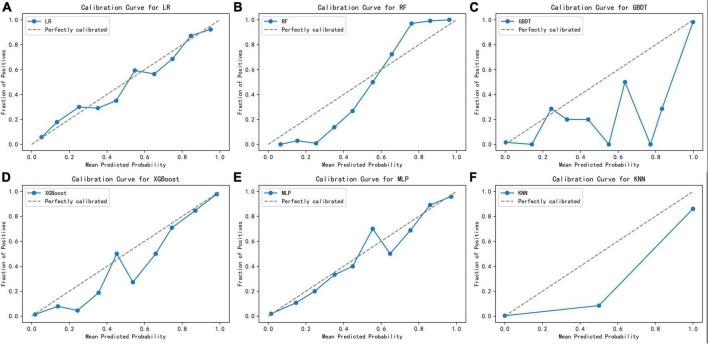
Calibration curves for the six learning models. **(A)** Logistic regression, **(B)** random forest, **(C)** gradient boosting decision tree, **(D)** XGBoost, **(E)** multilayer perceptron, **(F)** K-nearest neighbor.

Figure 2 displays an AUC visualization for ten-fold cross-validation. The AUC of the GBDT model was 0.971 (0.957–0.986), followed by the XGBoost model at 0.956 (0.939–0.973) and the RF at 0.955 (0.936–0.974). The probability curves for each model are displayed in Figure 3. The GBDT, XGBoost, and MLP models exhibited the least overlap and demonstrated a large separation between positive and negative events. These models revealed significant differences between patients who died and those who survived, indicating a higher capacity for discrimination. Figure 4 displays the calibration curves for each model, providing further quantification of this discrimination. The calculation of their calibration slopes in Table 2 confirms the improved effectiveness of the GBDT, XGBoost, and MLP models in differentiating patients with various outcomes. The analysis above demonstrates the usefulness of these three models in clinical decision making. Furthermore, Box plots of the six models are in Supplementary Figure 1 in Supplementary Material 2. Among all models, RF and XGBoost perform better in distinguishing positive and negative samples.

After assigning scores to each performance in turn, GBDT exhibited the highest prediction performance score (45 points), followed by XGBoost (36 points, Table 2). Given that the GBDT calibration curve oscillates between rising and falling values around the ideal curve and it performs mediocrely in distinguishing positive and negative samples Supplementary Figure 1 in Supplementary Material 2, XGBoost was chosen for additional model interpretation in this investigation.

**FIGURE 5:**
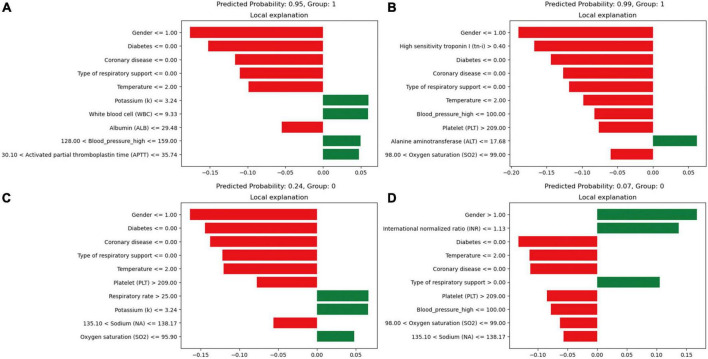
Local interpretable model-agnostic explanations (LIME) locally interpretable model agnostic interpretation map. Positive event: 1; Negative event: 0; Male: 1, Female: 2; **(A)** Deceased patients, true-positive cases; **(B)** deceased patients, true-positive cases; **(C)** surviving patients, true-negative cases; **(D)** surviving patients, true-negative cases. The picture presents the top 8 variables that had the greatest impact on survival or death from top to bottom. The length of the bar for each feature indicates the importance (weight) of that feature in making the prediction. A longer bar indicates a feature that contributes more to survival or death. Green bars indicate protective factors and red bars indicate risk factors. *x*-axis indicates the extent to which each predictor variable affects the final probability of a particular patient. The predicted probability of a patient’s death, as well as the actual outcome, is shown in each graphic caption.

Model interpretability, based on the XGBoost model, rates the variables and visually represents their contribution to the probability of death. Figure 5 presents four cases using the LIME validation set, including two death cases (Figures 5A, B) and two survival cases (Figures 5C, D). These charts showcase the top ten factors that have the greatest impact on patient survival or death and explain how these characteristics influence patient outcomes. Specifically, Figure 5A illustrates that male gender, absence of diabetes, absence of chronic pulmonary disease, use of non-invasive ventilation, and presence of low albumin levels (≤29.48 g/L) increase the risk of death. On the other hand, low potassium levels (≤3.24 mmol/L), normal white blood cell counts, normal systolic blood pressure values (128∼159 mmHg), and normal APTT (30.1∼35.74 s) reduce the risk of death. The comprehensive evaluation of this model predicted a probability of death of 0.95 for the patient in question and correctly classified them as deceased.

**FIGURE 6:**
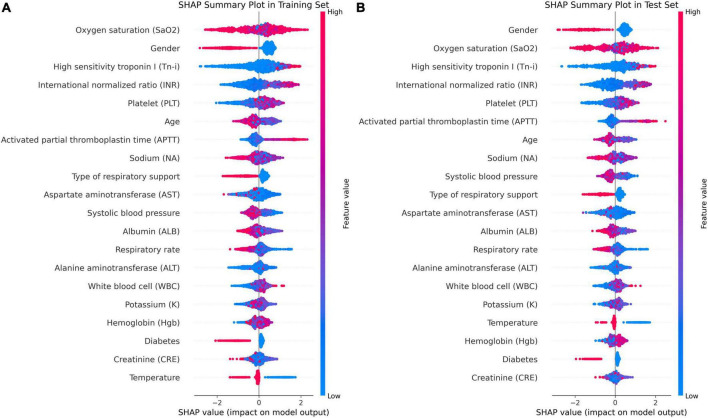
Importance ranking of SHAP variables based on the XGBoost model. **(A)** XGBoost SHAP graph on the training set. **(B)** XGBoost SHAP graph on the test set; Each line represents a feature, and the abscissa is the SHAP value. Red dots represent higher feature values, and blue dots represent lower feature values. In terms of Gender, red dots represents female.

In Figure 5D, factors such as female, INR value ≤1.13, and use of invasive ventilation were identified as reduce the risk of death in a patient. Conversely, the absence of diabetes, absence of chronic pulmonary disease and normal body temperatures, systolic blood pressure values, PLT, and sodium levels helped increase the risk of death. The combined evaluation of this model predicted a probability of death of 0.07 for the patient in question and correctly classified them as surviving. Meanwhile, Figures 6A, B demonstrate that gender, SaO_2_, Tn-i, INR, and PLT are the top five variables associated with death. The figures use a color scale, ranging from blue to red, to represent values from low to high. The axis at 0 serves as a critical divider: variables positioned to the left are considered protective factors, reducing the risk of death, while those on the right are risk factors, increasing the likelihood of death. For instance, an increase in Tn-i implies a higher risk of death.

**FIGURE 7:**
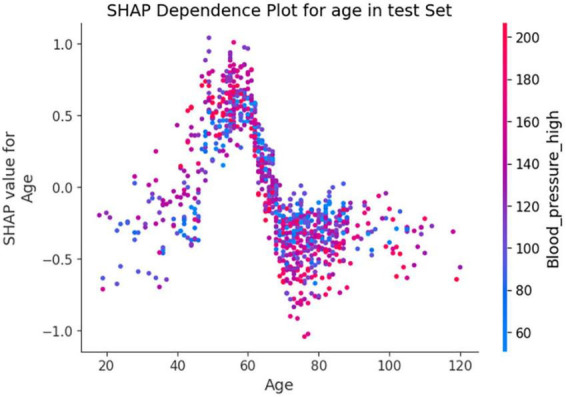
Shapley Additive exPlanations (SHAP) dependence plot based on the XGBoost model. SHAP values for specific features exceed zero, representing an increased risk of death.

In Figure 7, the SHAP dependence plot reveals that within the age group of 50 to 70 years, when systolic blood pressure exceeds 140 mmHg, SHAP values increase significantly and mainly fall within the positive value range. This suggests that hypertension patients in this age group face a higher risk of death from lung inflammation. However, after age 70, high systolic blood pressure seems to act as a protective factor against the risk of death from lung inflammation.

**FIGURE 8:**
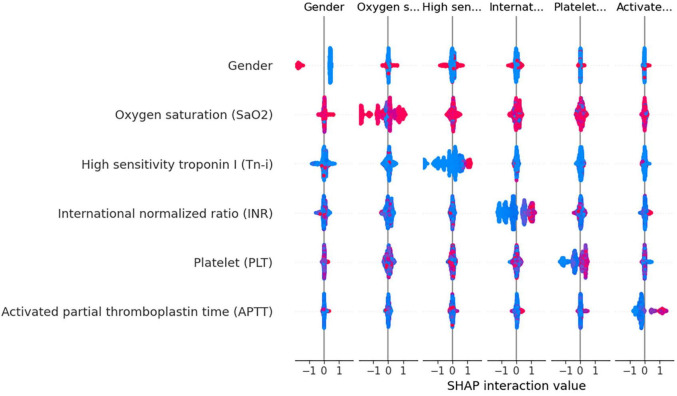
Variable interaction graph based on the XGBoost model. Red dots indicate higher values of a feature, while blue dots represent lower values. The red area on the plot signifies that both variables under consideration are registering high values simultaneously. when this interaction is observed on the right side of the SHAP plot, it correlates with an increased risk of death. Conversely, positions on the left side indicate a reduced risk. Specifically regarding the feature “Gender,” red dots denote female patients.

Using the XGBoost model, we explored the interactions among key variables and presented an interaction diagram for the first six variables (Figure 8). These charts display the interaction between different variables using the distribution of SHAP values. When the interaction between two variables is significant, their corresponding SHAP values are distributed at both ends of the graph. On the other hand, variables with minimal interactions tend to have SHAP values concentrated near zero.

Taking gender and high-sensitivity troponin as an example, as shown in Figure 8, the interaction between these two variables is evident. Areas with a SHAP value of 0 contain mostly blue values, indicating that these variables contribute relatively little to the model output, without significant interactions. In contrast, red values are mainly distributed at both ends of the SHAP value, suggesting that under a specific combination of gender and Tn-i levels, these two variables have a substantial impact on the model prediction. This analysis provides a deeper understanding of the model’s behavior.

**FIGURE 9:**
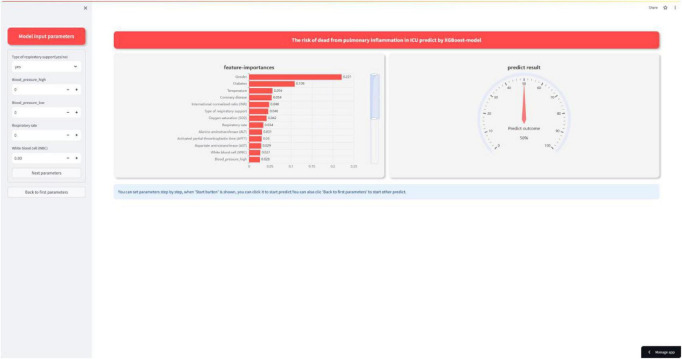
Interactive website.

In addition to the above analyses, we also developed a web-based calculator that can potentially be integrated with hospital information management systems for automated entry and recognition. The website is as follows: https://xgboost-project-app.streamlit.app/. On this website, users can simply input the actual measured values corresponding to the 25 variables mentioned above into the designated content boxes to trigger the model’s calculation and prediction process. Figure 9 provides an example diagram of the model home page. The XGBoost model can perform complex calculations and analyses based on these data.

## 4 Discussion

Research has indicated that patients admitted to ICU have a comparatively high death rate, which can range from roughly 15 to 40% (20–22). Previous studies have mainly focused on ICU-acquired infections (23) and the forecasting of COVID-19 pneumonia cases and fatalities (24, 25). Moreover, studies indicate that the Acute Physiology and Chronic Health Evaluation II (APACHE II) and the quick Sequential Organ Failure Assessment (qSOFA) scoring systems have a moderate predictive value for mortality among pneumonia patients admitted to the ICU (26, 27).

In practical clinical settings, doctors must undertake strenuous and complex efforts to consider a patient’s medical history, physical examination, and trends in vital signs. Accurate, reliable, quick, convenient, and rapid health assessments are crucial for doctors to make decisions that allow them to take appropriate emergency actions in a timely manner, especially for ICU patients. However, predicting the risk of death from pulmonary inflammation with machine learning techniques remains challenging. Therefore, we were able to effectively develop an interpretable machine learning model in this study to predict the in-hospital mortality probability of ICU patients with pulmonary inflammation. Our model excels in rapidly analyzing complex medical data to identify high-risk patients, thereby enabling timely intervention, optimizing resource allocation, and improving outcomes. It also supports personalized medical decision making, helping physicians develop optimal treatment plans for each patient and enhancing the overall efficiency of the healthcare system through precise risk assessments. In short, our model contributes to improved treatment effectiveness and medical resource utilization efficiency.

Prior to this study, previous research has predominantly focused on ICU-acquired infections and the progression of COVID-19 pneumonia, with an emphasis on mortality prediction. However, there has been a scarcity of interpretable machine learning methods tailored for lung inflammation mortality risk prediction. Our model addresses this gap by enabling clinicians to swiftly analyze complex medical data, thereby identifying patients at high risk. This facilitates timely interventions, optimizes the allocation of medical resources, and supports personalized treatment planning, enhancing both patient outcomes and the efficiency of medical care systems. The model’s interpretability ensures that clinicians can make informed decisions, thereby improving the overall effectiveness of patient management in critical care settings. Specifically, in our entire cohort, 7.19% (120/1668) of patients experienced early death. Notably, lung infections can progress to sepsis, the leading cause of infection-induced death. Table 3 compares our study to several others, where our study showed excellent performance in specific indicators. Based on the significance of SHAP variables, it was determined that gender, SaO_2_, Tn-i, INR, and PLT were the top five important variables associated with early death.

**TABLE 3:** Comparison with previous studies.

References	Region	Deaths (number of patients)	Disease	Performance
Jeon et al. (41)	Republic of Korea	27.3% (223/816)	Severe pneumonia	ACC: 0.822
				PRE: 0.860
				REC: 0.440
				AUC: 0.856
				Brier score: 0.120
Hu et al. (42)	United States	12.56% (1107/8817)	Sepsis	ACC: 0.895
				AUC: 0.884
Pan et al. (43)	China	47.2% (58/123)	COVID-19	ACC: 0.760
				Sensitivity: 0.667
				AUC: 0.913
				Youden index: 0.733
Wen et al. (44)	China	18.4% (41/223)	Hospital-acquired pneumonia	AUC: 0.863
This work	China	7.19% (120/1668)	Pneumonia	ACC: 0.889
				PRE: 0.871
				REC: 0.913
				AUC: 0.881
				Brier Score: 0.050
				Youden index: 0.947
				Calibration slope: 0.957

The INR value is a key indicator for measuring the activity of the coagulation system. It reflects the status of blood coagulation function and is an important indicator for evaluating liver dysfunction. In our study, we discovered that patients with pulmonary inflammation exhibited abnormal liver function indicators, such as altered levels of INR, albumin, and ALT. These abnormalities suggest a potential impairment in liver function. The liver serves as the primary organ responsible for the metabolism and detoxification processes in the human body. Consequently, even a modest decline in liver function can result in metabolic alterations, leading to the accumulation of toxins and worsening the disease’s systemic inflammatory response.

Studies have shown that lung inflammation leads to the release of numerous inflammatory mediators, which can, in turn, trigger immune-mediated liver damage, creating a harmful cycle (28). This suggests that when liver dysfunction causes an elevated INR, it impairs coagulation and indicates a weakened ability to respond to inflammation. For instance, studies have found that among patients with liver dysfunction, pulmonary inflammation is one of the most common infectious diseases. In viral pneumonia, the disease can cause cytopathic effects and damage to the endothelial cells, activating platelet and subendothelial aggregation, resulting in hypercoagulability (29, 30). At the same time, the pathogen recognition ability of the immune system and acquired immune system is strengthened, triggering the release of many inflammatory mediators, activating macrophages and T cells to clear viruses and kill infected cells. This not only causes a hypercoagulable state but also severe liver damage. This interaction is directly reflected in the observed increase in INR and exacerbation of liver function in patients with lung inflammation, making these indicators important in predicting a patient’s risk of death.

Elevated Tn-i is generally considered a biochemical marker of cardiomyocyte damage, reflecting the degree of damage to the heart muscle cells. In our study, the death group had significantly higher levels of Tn-i compared to the survival group. In cases of lung inflammation, especially severe ones, the heart may be indirectly affected. For example, severe lung infection can trigger a systemic inflammatory response, leading to an increase in inflammatory mediators in the blood, such as interleukin-2 (IL-2), IL-4, IL-6, IL-7, IL-18, and interferon- γ (31), and these mediators lead to cardiac dysfunction and structural damage (32). Specifically, these mediators can cause cardiac dysfunction and structural damage. Infections and inflammatory reactions may also increase the metabolic demand of the heart, and insufficient oxygen supply can further disrupt the metabolism of cardiomyocytes, increasing the risk of cell damage. Additionally, cell infiltration caused by the inflammatory response can lead to inflammatory damage to myocardial tissue and accelerate the release of troponin (33). Therefore, in the context of lung inflammation, elevated troponin is strongly associated with the risk of death.

In conclusion, the key features of the SHAP chart provide crucial insights into the progression and poor prognosis of pneumonia. Most of the indicators support our knowledge from clinical experience. By monitoring these indicators, medical personnel can gain valuable clues that may aid in the early detection of potential risks. This early recognition enables healthcare providers to swiftly implement appropriate interventions. As a result, this proactive approach can significantly enhance the clinical management of patients with pulmonary inflammation disease, ultimately improving their overall care and outcomes.

In the process of building a machine learning model, we utilized various methods for training and optimizing the model, such as the LR, RF, GBDT, XGBoost, MLP, and KNN algorithms. We initially focused on prediction probability plots, visually illustrating how well the model performs under different prediction probabilities. The areas where the curves overlap for positive and negative outcomes are particularly important because they indicate the level of uncertainty the model faces when predicting different outcomes. We noticed a significant overlap in the curves of the LR model, suggesting that the model struggled to distinguish between positive and negative results. This difficulty may be due to the model’s linear assumption of the data, which limits its performance.

Another crucial factor in interpreting the model’s predictions is the position of the peak on the predicted probability curve. A peak closer to 1 or 0 signifies higher confidence and accuracy in predicting a specific outcome. For example, the GBDT, XGBoost, and MLP models exhibited more concentrated peaks, indicating that these models can provide more accurate predictions when dealing with complex data structures. Furthermore, We conducted a thorough evaluation of multiple machine learning models to determine the most suitable one for deployment. This process can be particularly challenging when the performance metrics of the models are closely matched. To address this, we meticulously assessed each performance indicator, ranking the models from highest to lowest based on their scores. Our analysis revealed that the Gradient Boosting Decision Tree (GBDT) model achieved the highest overall score, closely followed by XGBoost.

Although the GBDT model exhibited strong performance across several metrics, its calibration curve showed significant deviations from the ideal. This was particularly noticeable within the prediction probability range of 30–80%. Moreover, the GBDT model’s predicted probabilities were consistently lower than the actual observed probabilities, indicating a potential underestimation issue. These observations necessitate a careful consideration of how the GBDT model’s calibration affects its reliability and accuracy in practical applications. On the other hand, although the XGBoost model slightly lags behind GBDT on some performance indicators, its built-in regularization measures and sensitivity to calibration optimization strategies make it more accurate in terms of probabilistic predictions. In the medical field, the requirements for the interpretability and probabilistic accuracy of predictions are particularly stringent. Taking these requirements into consideration, and after thoroughly evaluating the prediction probability curve, calibration curve, box plots, and other performance indicators such as accuracy, we selected the XGBoost model for further application and interpretation. This choice will facilitate its clinical use.

In the field of medical data mining and processing, machine learning has significant advantages over traditional statistical methods. It not only compensates for the limitations of linear models in handling complex data (34), but has also been widely used to develop prediction models for various diseases, such as lung cancer (35), liver cancer (36) and other chronic diseases (37, 38). However, machine learning models are often criticized for their “black box” characteristics in practical applications. This characteristic makes the internal decision making mechanism of the model difficult to intuitively understand, thereby affecting users’ trust and acceptance of the model (39). To address this issue, this study incorporates an efficient gradient-boosting machine learning framework: the XGBoost algorithm. It also utilizes a SHAP global variable importance map, LIME personalized explanations, and a web calculator to enhance the interpretability, accuracy, and transparency of the model. This enhancement aims to foster users’ trust in the model.

## 5 Limitations

First, this study was retrospective; therefore, we could not determine the severity of pneumonia. However, the study demonstrates that severe pneumonia comprises approximately 1.3% of all pneumonia patients (40). A more detailed discussion can aid in better disease management by considering the mortality rates of patients with mild/severe pneumonia. Second, this study has limitations regarding the population sample, as its relatively small number of participants may not adequately capture the potential diversity and heterogeneity within the patient population. Third, certain parameters and indicators are absent from the database, which hinders the analysis of factors such as organ failure sequential score and ventilator-specific parameters.

## 6 Conclusion

This study utilized XGBoost to develop a machine learning model for predicting the risk of death in ICU patients with pulmonary inflammation. The top five important variables were gender, oxygen saturation in arterial blood, high-sensitivity troponin-i, international normalized ratio, and platelets. To gain a deeper understanding of these variables in relation to mortality risk prediction, the LIME method was also used. This model aims to identify patients at a higher risk of early death to guide clinical decision making and improve patient care. However, further research is still needed to expand the sample size and conduct a stratified analysis of patients with mild and severe pneumonia in order to explore more practical treatments for patients with pulmonary inflammation.

## Data availability statement

The datasets presented in this study can be found in online repositories. The names of the repository/repositories and accession number(s) can be found below: https://physionet.org/content/icu-infection-zigong-fourth/1.1/.

## Ethics statement

The studies involving humans were approved by The Fourth People’s Hospital in Zigong. The studies were conducted in accordance with the local legislation and institutional requirements. The human samples used in this study were obtained from another research group. Written informed consent for participation was not required from the participants or the participants’ legal guardians/next of kin in accordance with the national legislation and institutional requirements.

## Author contributions

YZ: Conceptualization, Data curation, Formal analysis, Investigation, Methodology, Project administration, Software, Writing – original draft. DL: Data curation, Formal analysis, Methodology, Project administration, Software, Visualization, Writing – original draft. SL: Writing – original draft, Validation, Project administration, Formal analysis, Data curation, Conceptualization. LM: Conceptualization, Formal analysis, Resources, Validation, Visualization, Writing – review and editing.
