# Supplementary material for: Interpretability-based machine learning for predicting the risk of death from pulmonary inflammation in Chinese intensive care unit patients
Source: Front Med (Lausanne). 2024 Jun 12;11:1399527. doi: 10.3389/fmed.2024.1399527 (PMC11200536; doi:10.3389/fmed.2024.1399527)
Supplement: Supplementary file 1 [file Presentation_1.pdf]

Interpretability-based machine learning for predicting the risk of death from pulmonary inflammation in Chinese intensive care unit patients

Yihai Zhai; Danxiu Lin.; Siying Lv; Liqin Mo.

List of Supporting Information

Table S1. Hyper-parametric optimizationresults

Figure S1. Boxplots for six models

Table S1. Hyper-parametric optimization results

Model	Hyper-parameter space	Optimal hyper-parameter
LR	{'C': [0.001, 0.01, 0.1, 1, 10, 100], 'solver': ['newton-cg', 'liblinear', 'sag', 'saga']}	{'C': 1, 'solver': 'liblinear'}
RF	{'max_features':['sqrt','log2',None], 'min_samples_leaf':range(1,10,1), 'min_samples_split':range(2,10,1), 'n_estimators':range(30,300,5)}	{'max_features': 'sqrt', 'min_samples_leaf': 1, 'min_samples_split': 2, 'n_estimators': 100}
GBDT	{"learning_rate':[0.1,0.01,0.001], 'max_depth':[3,5,10], 'max_features':['sqrt','log2',None], 'min_samples_leaf':range(1,10,1), 'min_samples_split':range(2,10,1), 'subsample':[0.5,0.7,1.0, 'n_estimators':range(30,300,5)}	{'learning_rate': 0.1, 'max_depth': 10, 'max_features': 'log2', 'min_samples_leaf': 1, 'min_samples_split': 2, 'n_estimators': 290, 'subsample': 0.7}
XGBoost	{'learning_rate':[0.1,0.01,1], 'max_depth':range(1,10,1), 'min_child_weight':range(2,10,1), 'subsample':[0.5,0.7,1.0], 'colsample_bytree':[0.5,0.7,1.0], 'gamma':[0,1,5], 'n_estimators':range(30,300,5)}	{'colsample_bytree': 0.5, 'gamma': 0, 'learning_rate': 0.1, 'max_depth': 9, 'min_child_weight': 2, 'n_estimators': 270, 'subsample': 1.0}

MLP	{'activation':['relu','tanh'], 'alpha':[0.0001,0.001,0.01,0.1], 'hidden_layer_sizes':[(50,),(100,),(200,)], 'learning_rate':['constant','adaptive']}	{'activation': 'tanh', 'alpha': 0.0001, 'hidden_layer_sizes': (200,), 'learning_rate': 'constant'}
KNN	{'n_neighbors': range(1, 21), 'weights': ['uniform', 'distance'], 'metric': ['euclidean', 'manhattan'],	{'metric': 'manhattan', 'n_neighbors': 2, 'weights': 'uniform'}

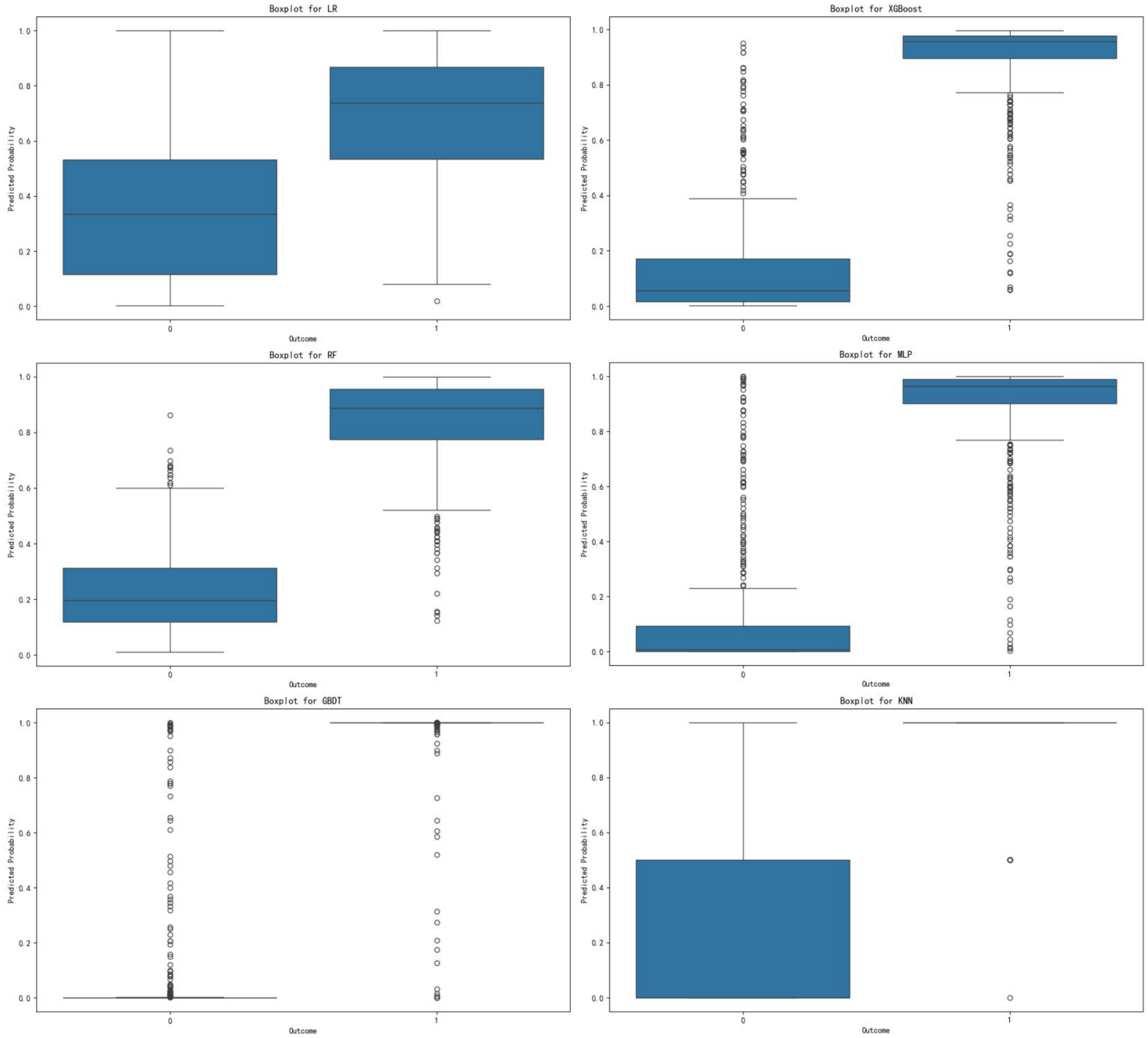

Figure S1. Boxplots for six models
